# Resting-state blink rate does not increase following very-light-intensity exercise, but individual variation predicts executive function enhancement levels

**DOI:** 10.1186/s40101-025-00390-x

**Published:** 2025-04-14

**Authors:** Ryuta Kuwamizu, Yudai Yamazaki, Naoki Aoike, Dongmin Lee, Hideaki Soya

**Affiliations:** 1 Laboratory of Exercise Biochemistry and Neuroendocrinology, Institute of Health and Sport Sciences, University of Tsukuba, Ibaraki, 305-8574 Japan; 2 Sport Neuroscience Division, Advanced Research Initiative for Human High Performance (ARIHHP), Institute of Health and Sport Sciences, University of Tsukuba, Ibaraki, 305-8574 Japan

**Keywords:** Spontaneous eye blink rate, Dopamine, Physical exercise, Cognitive function, Stroop task

## Abstract

**Background:**

Acute physical exercise, even at a very-light-intensity, potentiates prefrontal cortex activation and improves executive function. The underlying circuit mechanisms in the brain remain poorly understood, though we speculate a potential involvement of arousal-related neuromodulatory systems. Recently, our rodent study demonstrated that exercise, even at light-intensity, activates the midbrain dopaminergic neurons. Resting-state spontaneous eye blink rate is linked to brain-arousal neural circuits, and potentially to those modulated by dopaminergic system. We hypothesized that neural substrates linked to resting-state eye blink rate contribute to the cognitive impact of acute very-light-intensity exercise.

**Method:**

We analyzed data from a previous study with a renewed focus on resting-state eye blink rate. Twenty-four healthy young adults completed both 10 min of cycling (very-light-intensity exercise: 30% peak oxygen uptake) and rest conditions. Resting-state eye blink rate and Stroop task performance were measured before and after both exercise and resting control.

**Results:**

Results showed no significant differences in eye blink rate changes between conditions. However, correlation analyses revealed that exercise-induced changes in resting-state eye blink rate were significantly associated with individual variations in Stroop task performance enhancement.

**Conclusion:**

Very-light-intensity exercise does not elicit a consistent increase in eye blink rate after exercise. This finding does not support the involvement of a blink increase-linked neural substrate in enhancing executive function through very-light-intensity exercise. However, resting-state eye blink rate that is altered by exercise is predictive of executive function enhancement levels; this may serve as a novel contactless biomarker for predicting exercise benefits for brain health and cognition.

**Supplementary Information:**

The online version contains supplementary material available at 10.1186/s40101-025-00390-x.

## Background

An active lifestyle promotes brain health [1]. Acute and chronic exercise, even very-light-intensity exercise, can enhance cognitive health related to the prefrontal cortex [2, 3]. The ascending arousal system play an important role in prefrontal cortex cognitive function [4]. In considering neural dynamics induced by acute exercise for cognitive enhancement, we speculate the involvement of exercise-induced activation of the ascending arousal system, particularly brainstem catecholaminergic (dopaminergic (DA) and noradrenergic (NA)) neurons through our animal-to-humans translational research [5, 6]. However, the neurobiological mechanisms are elusive and useful biomarkers for this have not yet been identified in humans.

Eye-based measurements are economical and non-invasive, which is useful in exercise-cognition science [3, 7–9]. Brainstem arousal and DA agents modulate blink rate in monkeys and humans; therefore, resting-state spontaneous eye blink rate (rssEBR) has been discussed as a biomarker linked to DA-related behavior [10–13]. Human positron emission tomography (PET) studies show that striatal DA release affects rssEBR [12, 14, 15]. For example, rssEBR decreases in Parkinson's disease, restored by a DA precursor [16]. Although a more precise mechanistic understanding is needed [17], rssEBR remains a potential biomarker for exploring DA-regulated cognitive enhancement [18]. Our previous cross-sectional study showed that a higher baseline rssEBR is associated with higher aerobic fitness and superior executive function, suggesting that rssEBR may mediate a fitness-cognition link [9]. This finding leads to the hypothesis that rssEBR may both provide mechanistic insight into and provide a reliable, non-invasive biomarker for the impact of exercise on cognition, not only for the effects of chronic exercise, but also for acute exercise.

We aimed to explore whether acute very-light-intensity exercise increases rssEBR and, subsequently, whether increased rssEBR following exercise predicts prefrontal cognitive enhancement. Our previous research shows that very-light-intensity exercise improves executive function with left dorsolateral prefrontal cortex (l-DLPFC) activity [3]. We also showed that pupil dynamics, a measure of the NA-linked arousal system, predict, while exercising, enhanced executive function [3]. The current study builds on our previous research by analyzing additional facets of our previous data to examine the association between rssEBR and enhanced executive function.

## Materials and methods

**Fig. 1:**
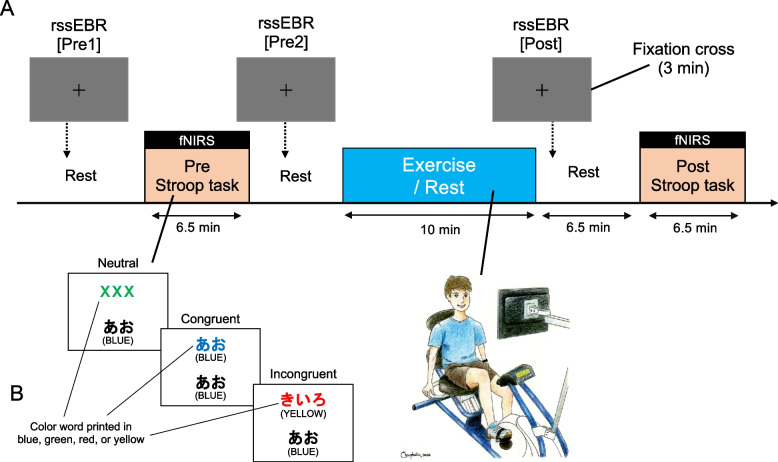
Summary of experimental paradigm. **A** Experimental paradigm flow. A color-word Stroop task was done before and after exercise/rest. Lateral prefrontal cortex activation was measured using functional near-infrared spectroscopy (fNIRS). **B** The Japanese version of the color-word-matching Stroop task. Participants responded with “yes” or “no” buttons, depending on whether the top font color matched the bottom color word or not. The task had 30 trials: 10 neutral, 10 congruent, and 10 incongruent, presented randomly. For neutral trials, the upper row contained crosses (XXX) in yellow, blue, green, or red, and the bottom row had color words written in black font. For congruent trials, the upper row had color words in colors that matched the meaning of the color word written in black in the bottom row. For incongruent trials, the upper row had color words in colors that did not match the meaning of the color word written in black in the bottom row to elicit cognitive conflict (i.e., Stroop interference)

We focused on rssEBR pre- and post-exercise, analyzing different facets of the data presented in Kuwamizu et al. [3]. Here we will briefly describe the original methods. Thirty-four healthy young adults, all native Japanese speakers, were recruited, of which 24 (3 females, mean age 21.7 years, SD = 1.2; 21 males, mean age 22.2 years, SD = 1.5) passed the screening (e.g., medication use, eye health condition, task comprehension) and participated in both 10 min of very-light-intensity exercise (30% $\dot{V\!}_{\text{O}_{2\text{peak}}}$) on a cycle ergometer and a resting control condition on separate days in a crossover design (Fig. 1). rssEBR was measured for 3 min before and after exercise.

### Resting-state spontaneous eye blink rate

rssEBR was measured before (Pre1 before Stroop test; Pre2 after Stroop test but before exercise) and immediately after (Post) exercise (EX) and control (CTL) conditions (Fig. 1). The focus was on resting-states before and after exercise to avoid the influence of body movements on eyelid activity, unlike other previous studies that focused on pupil diameter during exercise. Participants looked at a black fixation cross on a gray digital screen 70 cm away. rssEBR (blinks per minute) was recorded for 3 min using a camcorder set above the monitor. Two researchers independently assessed rssEBR, and the average of their scores was used [9]. We previously confirmed the validity of this camcorder count using a vertical electrooculogram recording method [9]. Correlations between researchers exceeded *r* = 0.99. Individual rssEBR was calculated by dividing the total number of blinks during the 3-min interval by 3. All data were collected by 6:00 pm because rssEBR can be less stable at night [9, 11]. Participants were not informed that their blinks would be measured to ensure natural blinking.

### Other variables

Executive function was assessed before and after exercise using a Stroop task (Fig. 1). Inverse efficiency scores (IES) were calculated as reaction time/accuracy. Stroop interference IES [incongruent IES–neutral IES] was calculated as inhibitory control [19, 20], the core component of executive function. Our previous study reported that very-light-intensity exercise significantly reduced Stroop interference compared to control. These measurements were used to explore rssEBR change relationships in the current analysis. For detailed methods and results, refer to Kuwamizu et al. [3]. Further exploratory analyses combining pupil diameter and l-DLPFC activity measured by functional near-infrared spectroscopy (fNIRS) are addressed in Supplementary material 2: Extended data.

## Results

Statistical analyses were performed using GraphPad Prism V9; significance was *P* < 0.05.

**Fig. 2:**
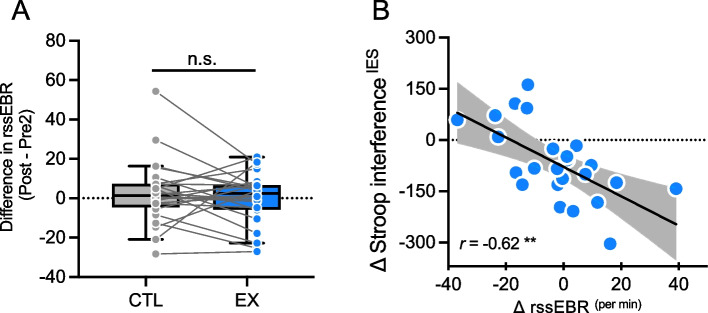
rssEBR change and the association between rssEBR change and Stroop interference. **A** The differences in rssEBR for both CTL and EX conditions. Pre2 was selected as the baseline to compare the immediate pre- and post-exercise states, ensuring a direct assessment of exercise-induced changes. The box-and-whisker plot is drawn in the Tukey manner. Line plots represent individual data. **B** Association between rssEBR ^EX (Post–Pre2)–CTL (Post–Pre2)^ and Stroop interference ^EX (Post–Pre)–CTL (Post–Pre)^ The line in the scatter plot represents linear regression, the band represents 95% confidence. **P < 0.01, ^n.s.^not significant

The mean ± SD of rssEBR (blinks/min) was 32.8 ± 19.3, 34.9 ± 19.2, and 35.0 ± 19.5 for the EX and 32.0 ± 19.4, 27.8 ± 17.1, and 30.5 ± 19.1 for CTL, measured at Pre1, Pre2, and Post, respectively. Figure 2A shows the change in rssEBR across conditions. Our main hypothesis focused on differences before and after exercise and rest conditions, so we compared the immediate pre-/post-differences (Post–Pre2) for both conditions using a paired *t*-test, which indicated no significant condition differences (*t*(23) = 0.79, *P* = 0.44) (Fig. 2A).

We then tested the relationship between rssEBR changes and Stroop task performance enhancement. To calculate the effect of very-light-intensity exercise on rssEBR, we computed the change in rssEBR as EX (Post–Pre2)–CTL (Post–Pre2). Similarly, for Stroop task performance, we analyzed the difference using EX (Post–Pre)–CTL (Post–Pre) [3]. The change of rssEBR ^EX(Post–Pre2)–CTL(Post–Pre2)^ significantly correlated with the reduction of Stroop interference ^EX(Post–Pre)–CTL(Post–Pre)^ (*r* (24) =  − 0.62, *P* = 0.001) (Fig. 2B).

## Discussion

We examined whether rssEBR, potentially involving brain DA modulation, explains the positive impact of acute very-light-intensity exercise on prefrontal executive function. Although very-light-intensity exercise did not elicit consistent rssEBR increase, there were significant associations between rssEBR variation and executive function enhancement post-exercise. These findings do not support the hypothesis that rssEBR-predicted neural substrates are the primary mechanism for cognitive enhancement. However, they do suggest that rssEBR change may predict executive function improvement levels following exercise.

These results do not support our hypothesis that very-light-intensity exercise increases rssEBR. Previous studies have produced mixed results; for example, a single bout of maximal aerobic exercise increased spontaneous EBR in adolescent boys with attention deficit hyperactivity disorder (ADHD) but not in girls with ADHD or typically developing children [21]. Other studies with smaller sample sizes (*n* = 16 or less) also failed to detect an increase in post-exercise blink rate, supporting our findings [22–24]. Here, we focused on rssEBR for 3 min immediately after exercise, but not during exercise. This was meant to exclude direct effects of physical and eye movements on blink rate. However, it is undeniable that DA upregulation may be eliminated immediately after exercise. Additionally, baseline rssEBR can vary depending on the study’s experimental conditions, such as visual stimuli. Although the baseline rssEBR was within the expected range and comparable to some previous data [12, 25], it tended to be higher overall compared to the average of previous studies [11], particularly on exercise condition days, which may reflect the motivational arousal to exercise. High baseline rssEBR may have negatively affected the detection of a potential exercise-induced increase in blink rates.

Interestingly, individual variations in rssEBR change highly correlated with improvements in executive function performance (Fig. 2B), which supports the hypothesis that rssEBR may provide mechanistic insight into and a useful biomarker of the impact of acute exercise on cognition. The direction of this correlation is similar to previous cross-sectional results showing that higher rssEBR is linked to better performance on the Stroop task [9]. Individual differences in exercise-induced DA release and receptor regulation may lead to variability in blink rate [12, 13], which, in turn, could predict post-exercise cognitive improvements.

Finally, the involvement of brain DA modulation in rssEBR remains uncertain [17]. Recently, it was reported that in humans, exercise induces the release of striatal endogenous DA correlated with the facilitation of simple decision-making reaction time [26]. Thus, to understand why rssEBR does not increase consistently with exercise, one speculative mechanism is the balance between D1 and D2 receptor activity [12] because their respective contributions to blink-rate change differ [27]. Other mechanisms, such as ocular factors, are also open for discussion.

## Conclusion

Acute very-light-intensity exercise does not consistently increase rssEBR, thereby not indicating the involvement of a blink increase-linked neural substrate in enhancing executive function through very-light-intensity exercise. However, rssEBR increased by exercise is predictive of individual executive function enhancement levels. This implies that blink rate may provide a novel non-invasive biomarker for predicting the cognitive benefits of exercise in humans.

## Supplementary Information

Supplementary Material 1: Sensitivity analysis.
Supplemental Material 2: Extended data.

## Data Availability

All data that support the findings of this study are available upon request from the corresponding authors within the limits set by the Institutional Ethics Committee of the University of Tsukuba, which ensures that personal information will not be disclosed.

## Abbreviations

ADHD: Attention deficit hyperactivity disorder
CTL: Control condition
DA: Dopamine
DLPFC: Dorsolateral prefrontal cortex
EX: Exercise condition
fNIRS: Functional near-infrared spectroscopy
IES: Inverse Efficiency Score
NA: Noradrenaline
PET: Positron emission tomography
rssEBR: Resting-State Spontaneous Eye Blink Rate
SD: Standard deviation
$\dot{V\!}_{\text{O}_{2\text{peak}}}$: Peak oxygen uptake
