# Supplementary material for: Resting-state blink rate does not increase following very-light-intensity exercise, but individual variation predicts executive function enhancement levels
Source: J Physiol Anthropol. 2025 Apr 14;44:10. doi: 10.1186/s40101-025-00390-x (PMC11995553; doi:10.1186/s40101-025-00390-x)
Supplement: Supplementary file 1 — Supplementary Material 1: Sensitivity analysis. Supplemental Material 2: Extended data. [file 40101_2025_390_MOESM1_ESM.pdf]

Supplementary material 1: Sensitivity analysis

Our main hypothesis focused on differences before and after exercise and rest conditions, so we compared the immediate pre/post differences (Post – Pre2) for both conditions using a paired *t*-test. This null result is supported by an optional analysis using repeated measures two-way ANOVA considering condition (EX, CTL) and time (Pre1, Pre2, Post), which showed no statistically significant effect of exercise intervention (interaction: time × condition, $F(1.50, 34.6) = 1.73$, $P = 0.20$; main effect of time, $F(1.59, 36.6) = 0.16$, $P = 0.80$; main effect of condition, $F(1.00, 23.00) = 5.33$, $P = 0.030$).

About the result of correlation analysis, Pre2 was selected as the baseline to compare the immediate pre- and post-exercise states. Regardless of the choice of baseline time point, this correlation was robust (rssEBR $^{\text{EX(Post–Pre1)} - \text{CTL(Post–Pre1)}}$, r (24) = -0.48, $P = 0.018$; rssEBR $^{\text{EX(Post)} - \text{CTL(Post)}}$, r (24) = -0.71, $P < 0.001$, respectively). The analysis accounting for the large inter-individual variance (eyeblink ratio of the Post-session relative to the Pre2-session) in baseline rssEBR (Pre2) yielded similar results (ssEBR $^{\text{EX(Post/Pre2)} - \text{CTL(Post/Pre2)}}$; r (24) = -0.53, $P = 0.0072$). These sensitivity analyses support the main finding shown in the main text.

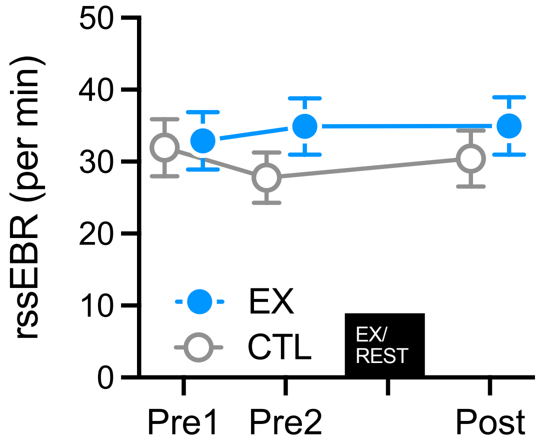

Figure S1. rssEBR for both CTL and EX conditions. Data are mean ± SE.

Supplemental material 2: Extended data

Here, we introduce the additional analysis on the associations between rssEBR change and neuroimaging and pupillometry data reported previously (Kuwamizu et al., 2023).

1. ***Prefrontal cortex activation measured by fNIRS***

The midbrain dopaminergic (DA) system plays a key role in executive functions through its influence on the prefrontal cortex and striatum [1]. In particular, it is well documented that the DA system regulates optimal noise control in the DLPFC [2]. These neural circuits are potential candidates for the neuromodulatory mechanisms underlying the beneficial effects of acute exercise on prefrontal executive function. Therefore, testing the association between exercise-induced task-related cortical activation and changes in rssEBR is valuable.

We previously reported that very-light-intensity exercise increases l-DLPFC activation [3]. This activation was measured using multichannel fNIRS (ETG-7000; Hitachi Medical Corporation, Tokyo, Japan). fNIRS measured prefrontal cortical activation by analyzing changes in oxygenated hemoglobin (oxy-Hb). ROIs were set in the left and right dorsolateral prefrontal cortex (DLPFC), ventrolateral prefrontal cortex, and frontopolar areas. Oxy-Hb changes due to Stroop interference (Incongruent task – Neutral task) were calculated. See our previous studies for detailed methodology [3,4].

Here, we conducted a correlation analysis similar to that for Stroop task performance. As results, there are a weak positive correlation between exercise-enhanced task-related l-DLPFC activation change $^{\mathrm{EX(Pos–Pre)\ –\ CTL(Post–Pre)}}$ and rssEBR change, but it was not significant (r (24) = 0.27, P = 0.21). Given that this dataset did not originally show a strong correlation between l-DLPFC activation levels and individual executive function enhancement [3], this outcome is understandable. These results suggest that the increased task-related prefrontal activation alone cannot explain why an increase in rssEBR predicts improvements in individual executive function. The striatal DA system may also contribute to executive function by networking with the DLPFC [1]. It is possible that the functional connectivity of the l-DLPFC with striatum via DA system plays a role in enhancing executive function [5]. These unexplored mechanisms warrant further investigation in future studies.

2. ***Exploratory analysis of pupil diameter and blink rate changes with exercise***

Previous research found that 10 minutes of very-light-intensity exercise (30% $\dot{V}\mathrm{o}_{2\mathrm{peak}}$) increased pupil dilation and reduced Stroop interference [3]. The degree of pupil dilation during exercise predicted Stroop performance improvements, supporting the hypothesis that pupil-linked mechanisms, possibly involving locus coeruleus (LC) activation, enhance cognitive function. In the present analysis, we show that exercise did not increase blink rate, but that changes in blinking after exercise correlated with improvements in Stroop task performance. This suggests a partial involvement of neural

substrates linked to blinking. Confirming the relationship between pupil changes and blinking changes due to exercise is crucial for expanding upon our previous findings and current insights. Therefore, we conducted exploratory analyses to discuss the relationship between pupil dynamics during exercise and changes in blink rate after exercise, as well as their respective predictions of cognitive task performance improvement due to exercise. This additional exploratory analysis aims to provide further insights into the mechanisms by which exercise influences executive function and to evaluate whether blink measurements could serve as an additional valuable biomarker for predicting the cognitive impact of exercise.

Additional exploratory analyses investigated the associations between rssEBR, pupil dilation, and Stroop task performance using correlation and multiple regression analyses. First, the change in sEBR $^{\text{EX(Post–Pre2) – CTL(Post–Pre2)}}$ positively correlated with pupil diameter changes before and after exercise/rest $^{\text{EX(Post–Pre2) – CTL(Post–Pre2)}}$ (r (24) = 0.52, P = 0.0092) and pupil diameter change before and during exercise $^{\text{EX([mean of Ex1 and Ex2]–Pre2) – CTL([mean of Ex1 and Ex2]–Pre2)}}$ (r (24) = 0.43, P = 0.037)(Fig.S3). Next, to examine the explanatory power of pupil dilation and changes in rssEBR for the reduction in Stroop interference due to very light-intensity exercise, we conducted a multiple regression analysis. This showed that both ΔrssEBR $^{\text{EX(Post–Pre2) – CTL(Post–Pre2)}}$ and Δpupil diameter significantly explained the effect of exercise on Stroop interference (ΔrssEBR $^{\text{EX(Post–Pre2) – CTL(Post–Pre2)}}$, β = -0.43; Δpupil diameter $^{\text{EX([mean of Ex1 and Ex2]–Pre2) – CTL([mean of Ex1 and Ex2]–Pre2)}}$, β = -0.44)(Table S1). It is important to note that, unlike pupil diameter measurements, rssEBR was not recorded during exercise.

Individuals who exhibited pupil dilation during exercise also had an increase in blink rate. This aligns with the interconnected activation of the DA and noradrenergic (NA) systems, which are closely linked [6]. Changes in blink rate and pupil dilation both significantly contributed to predicting reduced Stroop interference, suggesting their role in identifying enhanced executive function through very-light-intensity exercise. This indicates that the activation of both the NA system (possibly reflected by pupil dilation) and the DA system (possibly reflected by increased blink rate) plays a crucial role in the cognitive benefits observed from such exercise.

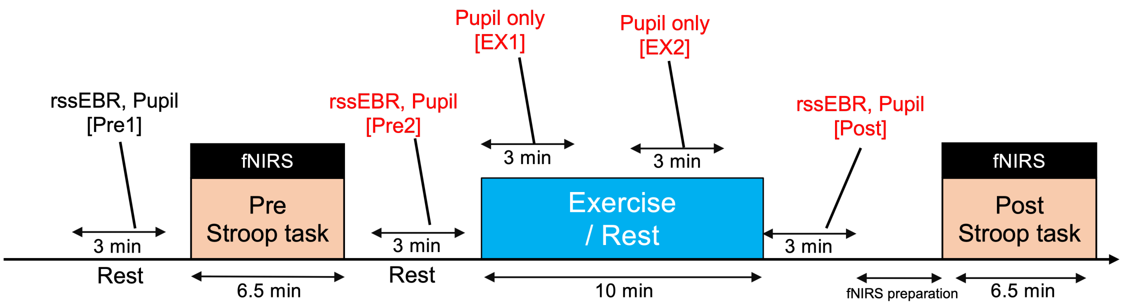

Figure S2. Experimental paradigm outline showing timing of pupillometery and blink measurements. Measurements at the times shown in red were the main data used in the analysis

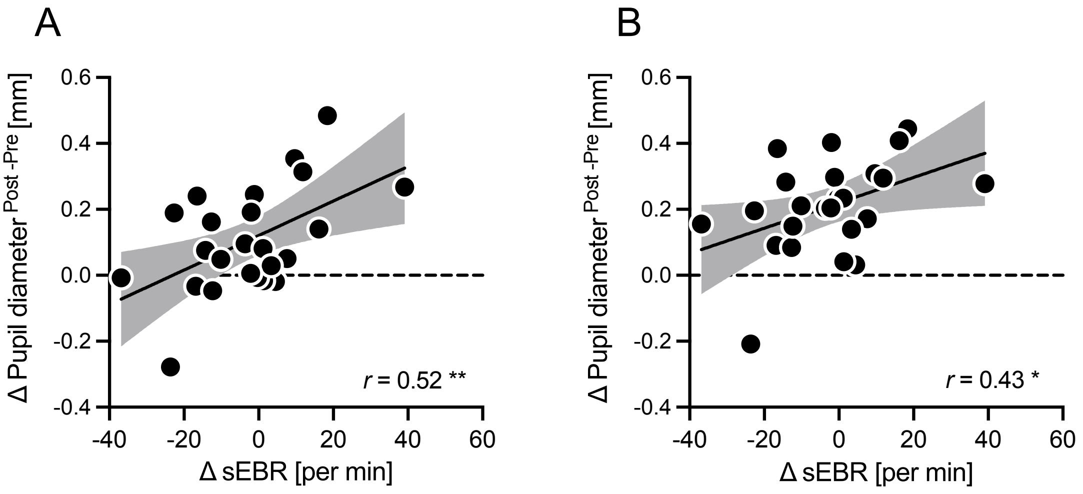

Figure S3. (A) Correlation between ΔsEBR (EX (Post – Pre2) – CTL (Post – Pre2)) and ΔPupil diameter (EX (Post – Pre2) – CTL (Post – Pre2)). (B) Correlation between ΔsEBR (EX (Post – Pre2) – CTL (Post – Pre2)) and ΔPupil diameter (EX ([mean of Ex1 and Ex2] – Pre2) – CTL ([mean of Ex1 and Ex2] – Pre2) – Pre2)). The scatter plot lines represent linear regression, and the bands represent 95% confidence. *$P < 0.05$, **$P < 0.01$

Table S1. Results of multiple regression analysis

	R^2	Adjust R^2	B	*SE* B	*t* value	*P* value
Model	0.547	0.504				< 0.001***
(Intercept)			-3.869	31.876	-0.121	0.9046
ΔPupil $^{EX\text{-}Pre2}$			-339.149	125.096	-2.711	0.0131*
ΔrssEBR$^{Post\text{-}Pre2}$			-2.998	1.122	-2.673	0.0142*

Note: R^2: coefficient of determination; B: unstandardized regression coefficient; *SE* B: standard error. *P < 0.05, ***P < 0.001. Analysis performed in R 4.3.2 using the R package “lm.beta”.

References

1. Cools R. Chemistry of the adaptive mind: Lessons from dopamine. Neuron. 2019;104:113–31.
2. Cools R, Arnsten AFT. Neuromodulation of prefrontal cortex cognitive function in primates: the powerful roles of monoamines and acetylcholine. Neuropsychopharmacology. 2022;47:309–28.
3. Kuwamizu R, Yamazaki Y, Aoike N, Hiraga T, Hata T, Yassa MA, et al. Pupil dynamics during very light exercise predict benefits to prefrontal cognition. Neuroimage. 2023;277:120244.
4. Damrongthai C, Kuwamizu R, Yamazaki Y, Aoike N, Lee D, Byun K, et al. Slow running benefits: Boosts in mood and facilitation of prefrontal cognition even at very light intensity. bioRxiv. 2024. https://www.biorxiv.org/content/10.1101/2024.01.29.575971v1.abstract
5. Nagano-Saito A, Leyton M, Monchi O, Goldberg YK, He Y, Dagher A. Dopamine depletion impairs frontostriatal functional connectivity during a set-shifting task. J Neurosci. 2008;28:3697–706.
6. Ranjbar-Slamloo Y, Fazlali Z. Dopamine and Noradrenaline in the Brain; Overlapping or Dissociate Functions? Front Mol Neurosci. 2019;12:334.
